# Quasi‐Newtonian Environmental Scanning Electron Microscopy (QN‐ESEM) for Monitoring Material Dynamics in High‐Pressure Gaseous Environments

**DOI:** 10.1002/advs.202001268

**Published:** 2020-08-18

**Authors:** Jinlong Zhu, Lenan Zhang, Xiangyu Li, Kyle L. Wilke, Evelyn N. Wang, Lynford L. Goddard

**Affiliations:** ^1^ Department of Electrical and Computer Engineering University of Illinois at Urbana‐Champaign Urbana IL 61801 USA; ^2^ Department of Mechanical Engineering Massachusetts Institute of Technology Cambridge MA 02139 USA

**Keywords:** environmental scanning electron microscopy, high‐pressure gaseous chamber, material dynamics, mechanical work, Newtonian, scattering force

## Abstract

Environmental scanning electron microscopy (ESEM) is a powerful technique that enables imaging of diverse specimens (e.g., biomaterials, chemical materials, nanomaterials) in a hydrated or native state while simultaneously maintaining micro‐to‐nanoscale resolution. However, it is difficult to achieve high signal‐to‐noise and artifact‐free secondary electron images in a high‐pressure gaseous environment due to the intensive electron‐gas collisions. In addition, nanotextured substrates can mask the signal from a weakly scattering sample. These drawbacks limit the study of material dynamics under extreme conditions and correspondingly our understanding in many fields. In this work, an imaging framework called Quasi‐Newtonian ESEM is proposed, which introduces the concepts of quasi‐force and quasi‐work by referencing the scattering force in light–matter interactions, to break these barriers without any hardware changes. It is shown that quasi‐force is a more fundamental quantity that has a more significant connection with the sample morphology than intensity in the strongly scattering regime. Experimental and theoretical studies on the dynamics of droplet condensation in a high‐pressure environment (up to 2500 Pa) successfully demonstrate the effectiveness and robustness of the framework and that the overwhelmed signal of interest in ESEM images can be reconstructed through information stored in the time domain, i.e., frames captured at different moments.

## Graphical abstract

This work introduces an electron imaging framework called quasi‐Newtonian environmental scanning electron microscopy (ESEM) that outperforms conventional ESEM in characterizing materials dynamics in high‐pressure gaseous environments.

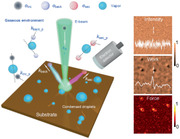

Environmental scanning electron microscopy (ESEM) has emerged as a powerful tool to characterize a diversity of samples including both wet and insulating materials in a straightforward manner, that is, no significant specimen preparation or metallic layer coating is required.^[^ ^1^, ^2^, ^3^ ^]^ Such an extraordinary feature has boosted the applications of ESEM in many fields such as colloidal dispersions,^[^ ^4^, ^5^, ^6^, ^7^ ^]^ chemical reactions,^[^ ^8^, ^9^, ^10^ ^]^ biological specimen,^[^ ^11^, ^12^, ^13^ ^]^ phase changes,^[^ ^14^, ^15^, ^16^ ^]^ micromechanics,^[^ ^17^ ^]^ and complex fluids.^[^ ^18^ ^]^ Generally, the success of ESEM relies heavily on the participation of gas molecules in the imaging process: electrons that escape from non‐conducting sample surfaces travel through the gas and produce a cascaded amplification of electrons by ionizing the gas molecules, resulting in positive ions that can drift down to the sample and thus compensate the accumulated surface charges.^[^ ^1^, ^19^ ^]^ However, the injection of gas vapor molecules in the chamber also poses a serious issue with image quality when the operation pressure is relatively high (e.g., ≈1000 Pa).^[^ ^1^ ^]^ As shown in **Figure** **1**a, the random and large‐angle collisions between electrons (incident, backscattered, and secondary electrons) and gas vapor molecules deflect the electrons from their original paths, and thus lower the spatial resolution and degrade the image contrast. This challenge cannot be addressed by simply decreasing the total travel distance for electrons because the large‐angle scattering of electrons can still happen at a position very close to the sample surfaces in a high‐pressure environment. It was demonstrated that the pressure limit of ESEM can be elevated above 2000 Pa, but an instrument‐level modification of the aperture holder is required.^[^ ^19^, ^20^ ^]^ In addition, a separate challenge arises for a sample with a low scattering cross‐section (SCS; for instance, water droplets) that is mounted on top of a rough substrate. When the characteristic length scale of the roughness is comparable to that of the sample, scattering from the random nanotexture can easily overwhelm the desired signals. This substrate effect is difficult to address through instrument‐level modifications or conventional image‐processing techniques because the random nanotexture behaves as “a second sample” rather than a typical additive noise source or a background perturbation. Meanwhile, a transport‐of‐intensity‐equation‐based phase reconstruction technique,^[^ ^21^ ^]^ as an alternative to conventional intensity‐based imaging, has been applied into electron microscopy.^[^ ^22^, ^23^, ^24^ ^]^ Significant enhancement of ESEM image quality in high‐pressure conditions (2500 Pa) was demonstrated very recently using phase retrieval;^[^ ^25^ ^]^ however, this technique is inherently sensitive to systematic errors in the raw ESEM images, such as a pixel with zero intensity or a line with an intensity discontinuity. The phase reconstruction process may fail due to the propagation of these types of local errors under the global operator of the transport‐of‐intensity equation (see the Principle Section, Supporting Information). In addition, the physical meaning of the reconstructed phase is still elusive, leading to fundamental challenges toward fully quantitative ESEM imaging using phase retrieval.

**Figure 1:**
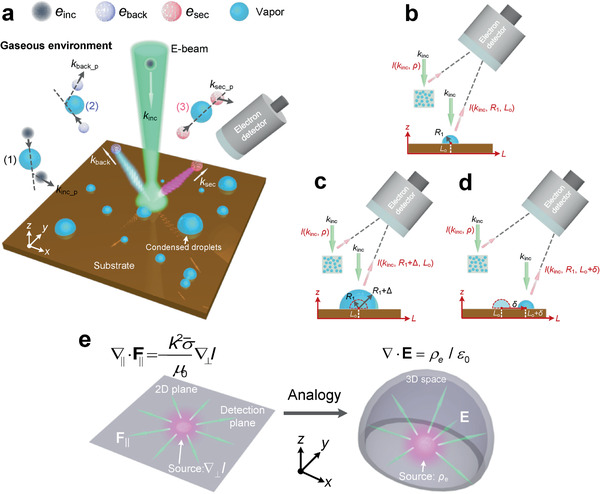
Schematic diagrams of the electron scattering in ESEM and the physical model of QN‐ESEM. a) Representative channels of electron‐matter collisions in the condensation experiment using a conventional ESEM system. Electrons interact with a condensed droplet under its three states, that is, b) original, c) expanded, and d) shifted ones. The temperature and pressure of the background gaseous environment are assumed to be constant. e) The governing equation in the proposed QN‐ESEM and its mathematical analogy to Gauss's law.

In this work, we demonstrate a new ESEM imaging framework called quasi‐Newtonian environmental scanning electron microscopy (QN‐ESEM), which is built upon the concepts of reconstructing quasi‐work and quasi‐force maps by referencing the optical virtual work and scattering force.^[^ ^26^, ^27^, ^28^, ^29^, ^30^ ^]^ To demonstrate the simplicity, robustness, and effectiveness of QN‐ESEM, dynamic imaging experiments of low‐SCS condensing droplets from backgrounds consisting of a high‐pressure gaseous chamber and random nanotextured substrates were performed. We show the key to simultaneously breaking the two barriers (large‐angle scattering due to electron–gas and electron–nanotexture interactions) in conventional ESEM is the time‐lapse information hidden in each frame that captures the dynamics of the sample. We utilize the fact that the raw ESEM images of a dynamic sample obtained at different times are not mathematically and physically independent. This insight facilitates defect‐free high‐resolution reconstruction of tiny objects from conventional ESEM experiments without hardware modifications.

Figure 1a shows three representative types of electrons, that is, the incident electrons (denoted by *k*_inc_), the backscattered electrons (denoted by *k*_back_), and the secondary electrons (denoted by *k*_sec_), existing in the ESEM chamber. Consequently, three types of undesired electrons exist (represented by *k*_inc_p_, *k*_back_p_, and *k*_sec_p_, respectively, where the subscript “p” denotes the water molecules or particles in the chamber) after the electron–gas molecule interactions. These electrons are believed to be the primary cause of image quality deterioration because of large‐angle collisions in conventional ESEM under high‐pressure environments.^[^ ^1^ ^]^ We consider a single condensed droplet located at *L*_0_ with a radius *R*_1_, whose scattering intensity *I *(*k*_inc_, *R*_1_, *L*_0_) is a function of incident beam, radius, and position (Figure 1b). For an isolated, stable, and adiabatic chamber with a given gaseous pressure, the density of the gas *ρ* is a constant according to the ideal gas law. This indicates that theoretically the statistically macroscale behaviors of background scattering intensity noise (denoted by *I *[*k*_inc_, *ρ*]) arising from the electron beam–gas interactions is time‐invariant. If the droplet grows in size to *R*_1_ + Δ or translates from *L*_0_ to a new position *L*_0_ + *δ*, the scattering from the droplet changes but the noise from the background gas does not (Figure 1c,d). For this reason, it is possible to use sequential time‐resolved ESEM frames to reduce the background scattering noise caused by electron beam–gas molecule interactions through finite difference method. However, this method has limited improvement for resolution or contrast because in practice the dynamic growth of droplets also perturbs the gaseous environment, and more importantly, the trajectories of the backscattered and secondary electrons after large‐angle collisions vary with the droplet growth. Therefore, conventional intensity difference maps cannot properly eliminate the strong electron‐gas scattering effects.

To further improve the performance of ESEM, we extend the concept of optical scattering force and virtual work to electron microscopy. In the optical domain, we previously showed these quantities are fundamentally more sensitive to weak scattering events than typical intensity maps.^[^ ^26^ ^]^ Here, we present the method for reconstructing the quasi‐force and quasi‐work in the ESEM domain. The quasi‐force and quasi‐work are quantities derived by referencing the elastic photon‐matter collision, but they are not true electron‐induced force and work because the electron‐matter collision is inelastic and the corresponding governing equations are not Maxwell's equations. This is the reason we use the prefix “quasi.” However, we will show in the following that the quasi‐force is a physics‐relevant quantity because it is directly relevant to the geometrical morphology of samples under investigation. Although the “quasi‐force” is not a fully quantitative measurand to describe the complex electron scattering mechanisms, it utilizes the high intrinsic sensitivity of the scattering force and the polychromatic wave to improve the imaging performance.^[^ ^21^, ^26^ ^]^ Moreover, quasi‐work surpasses conventional measurands in electron microscopy such as intensity and phase in terms of robustness and sensitivity. Following the reconstruction of the optical scattering force, we derived an analogous equation for the electrons in the ESEM

(1) $$\begin{array}{c} \nabla_{∥}\cdot F_{∥}=-\frac{k^{2}\bar{\sigma }}{\mu_{0}}\nabla_{⊥}I \end{array}$$

where **F**_∥_ is the quasi‐force parallel to the plane of detection, *k* is the modulus of wave vector **k**, $\bar{\sigma }$ is the equivalent SCS, *μ*_0_ is the permeability of vacuum that arises from an optical material system, *I* is the detected intensity of electrons, ∇_⊥_ denotes the longitudinal partial derivative (perpendicular to the detection plane), and ∇_∥_ · is the transverse divergence operator. *k* is a function of electron wavelength, and $\bar{\sigma }$is determined by the electron wavelength, electron beam energy, molecular ionization energy, and molecular potential, where the electron wavelength and energy are determined by the applied accelerating voltage.^[^ ^3^ ^]^ The term $\frac{k^{2}\bar{\sigma }}{\mu_{0}}\nabla_{⊥}I$ acts as a source for the field **F**_∥_. If the sample morphology evolves temporally, this source, and thus the field **F**_∥_, can be estimated as the central difference time derivative from a sequence of ESEM images.^[^ ^25^ ^]^ Because the collisions are inelastic, the detected electrons contain a mixture of electrons with various de Broglie wavelengths; thus, the detected signal is polychromatic, for which reason **F**_∥_ can be understood as a quantity that measures the average quasi‐force parallel to the detection plane. Because *k*, $\bar{\sigma }$, and *μ*_0_ behave as scaling constants (see Section S1, Supporting Information), the polychromaticity of the detection signals only changes the absolute values of **F**_∥_, but does not affect the overall distribution of the quasi‐force maps.^[^ ^26^ ^]^ Moreover, by analogy with Gauss's law of 3D electrostatics (Figure 1e), Equation (1) depicts the distribution of **F**_∥_ due to ∇_⊥_ *I* in the detection plane: positive (negative) ∇_⊥_ *I* is a source (sink) for field lines for **F**_∥_, and the flux of field lines for **F**_∥_ is independent of the surrounding curve in the 2D plane provided that the total “sources” enclosed is fixed. Hence, **F**_∥_ provides physical insights into electron–matter interactions and provides a more straightforward measure than conventional phase imaging to reconstruct the morphology of the samples in a strongly scattering regime. Based on the relationship between quasi‐work and quasi‐force, Equation (1) can be expressed into a more concise form

(2) $$\begin{array}{c} \Delta_{∥}w_{∥}=-k\nabla_{⊥}I \end{array}$$

where the product of the reconstruction‐irrelevant constants *μ*_0_ and $\bar{\sigma }$ is set to unity. Here, *w*_∥_ is the normalized quasi‐work, which generates the normalized quasi‐force, that is, **f**_∥_ = ∇_∥_ *w*_∥_. The reason we call **f**_∥_ normalized quasi‐force (and *w*_∥_ normalized quasi‐work) is that it is computed by normalizing **F**_∥_ (and the quasi‐force $W_{∥}=F_{∥}\cdot \vec{s}$; where $\vec{s}$ denotes the distance) with constant terms (see the Principle Section, Supporting Information). Because the intensity *I* is a detectable physical quantity in any conventional ESEM, *w*_∥_ can be reconstructed by numerically solving Equation (2) using the finite difference method.^[^ ^21^ ^]^

To validate the quasi‐work and quasi‐force reconstruction, we first carried out dropwise condensation experiments in an ESEM chamber (EVO 50, Zeiss) at a saturation vapor pressure of 1300 Pa, which is a high‐pressure condition compared to many ESEM‐based condensation experiments.^[^ ^31^, ^32^, ^33^ ^]^ The top three images in **Figure** **2**a correspond to three intensity maps captured at three successive frames, in which the growth dynamics of the droplets can hardly be resolved. The normalized first‐order difference between the last two frames (*I_R_*_1+2Δ_ and *I_R_*_1+Δ_), shown in the sub‐figure at the bottom of Figure 2a, cannot eliminate the random background scattering, as has been discussed previously. In contrast, the normalized quasi‐work map *w*_∥_ recovers the size and distribution of each droplet with a significantly high contrast (Figure 2b). For the ESEM used in our experiment, the secondary electron detector is mounted at an inclined angle with respect to the normal of the substrate, leading to a shadow region behind each droplet (tail side of the dashed white arrow in Figure 2b). The normalized quasi‐force map **f**_∥_ for the same area of interest is shown in Figure 2c. The in‐plane quasi‐force distribution due to the selected droplet behaves like an elliptical donut (the dashed white box in Figure 2c). Here, we discuss the significance of the normalized quasi‐force map at both the fundamental and application levels. The magnitude of **f**_∥_ (i.e., |**f**_∥_|) depends on the morphology of the droplets. Figure 2d shows a projection of the droplet geometry along the detection direction. We postulate that the statistical average of the real scattering force $f_{\sec}\left(\vec{r}\right)$ induced by secondary electrons at a position $\vec{r}$ is perpendicular to the liquid–vapor interface (Figure 2d). This seems reasonable because of the spherical symmetry of the droplet. The postulate also ensures the total horizontal force acting on the entire droplet is zero. The projection of $f_{\sec}\left(\vec{r}\right)$ on the detection plane $f_{\mathrm{proj}}\left(\vec{r}\right)$ can be written as

(3) $$\begin{array}{c} \left|f_{\mathrm{proj}}\left(\vec{r}\right)\right|=\left|f_{\sec}\left(\vec{r}\right)\cdot \vec{u}\right| \end{array}$$

where $\vec{u}$ is the unit vector parallel to the detection plane. $|f_{\mathrm{proj}}\left(\vec{r}\right)|$ reaches its maximum at point A of Figure 2d. When $f_{\sec}\left(\vec{r}\right)$is perpendicular to the detection path, $|f_{\mathrm{proj}}\left(\vec{r}\right)|$ becomes zero as shown in point B of Figure 2d. Therefore, $|f_{\mathrm{proj}}\left(\vec{r}\right)|$ is a function of the interface profile. Interestingly, we found that the magnitude of the cross‐sectional profile of the measured normalized quasi‐force, $|f_{∥}\left(\vec{r}\right)|$, matches well with that of the projection, $|f_{\mathrm{proj}}\left(\vec{r}\right)|$. Figure 2e shows the distribution of $|f_{\mathrm{proj}}\left(\vec{r}\right)|$ (red solid line) along the path (blue line in the inset of Figure 2e) predicted by Equation (3) with a spherical morphology of the droplet. The experimentally reconstructed $|f_{∥}\left(\vec{r}\right)|$ from QN‐ESEM crossing the same droplet (blue line in the inset of Figure 2e) shows good agreement with $|f_{\mathrm{proj}}\left(\vec{r}\right)|$, indicating that $|f_{∥}\left(\vec{r}\right)|$ is a good indicator for the interface profile and can be useful for the reconstruction of sample morphology in the strongly scattering regime. Note that although the phase‐based enhanced ESEM approach can also achieve much better image quality compared with the raw intensity map (Figure 2a),^[^ ^25^ ^]^ the relationship between the quasi‐phase and sample morphology is subtle because the strong inelastic scattering of polychromatic electrons reduces the coherence, resulting in particular challenges for fully quantitative analysis. The vertical error bar of $|f_{∥}\left(\vec{r}\right)|$ shown in Figure 2e was determined from the standard deviation of the background random scattering, while the horizontal error bar corresponds to the pixel size of QN‐ESEM.

**Figure 2:**
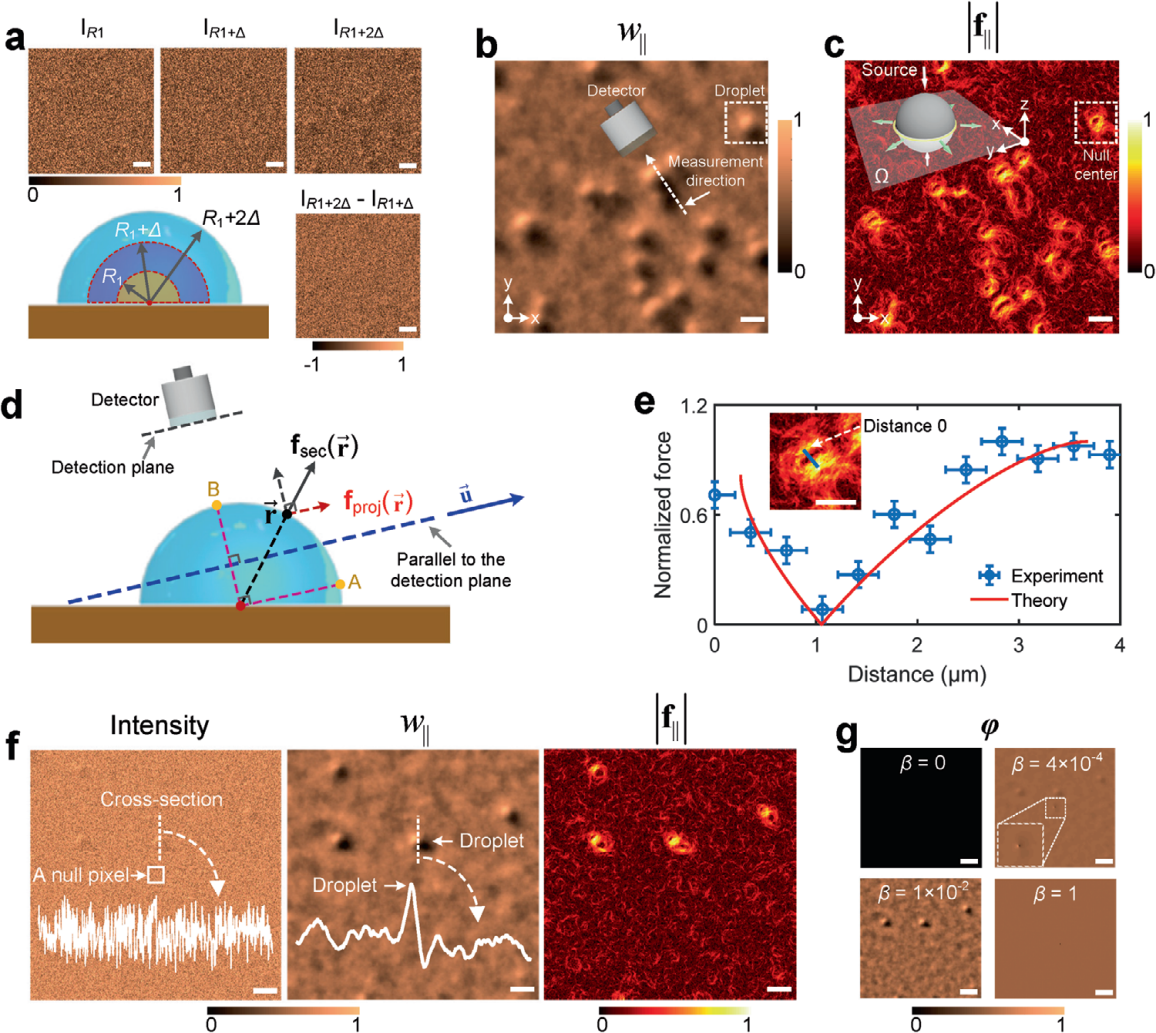
Features of normalized quasi‐work and quasi‐force imaging in QN‐ESEM and a comparison with the phase reconstruction method. a) Intensities captured at three states for droplets as they grow from *R*_1_ to *R*_1_+2Δ. The difference (*I*_R1+2Δ_ – *I*_R1+Δ_) is presented at the bottom right image. The reconstructed normalized b) quasi‐work and c) quasi‐force maps. The projection direction of the electron beam is evident from the shadow of each droplet. The inset in (c) illustrates the fact that the normalized quasi‐force is primarily concentrated at the border of a homogenous and isotropic object. d) Schematic showing the theoretical model of ESEM‐based detection. e) Experimental normalized quasi‐force curve and the corresponding theoretical prediction. The inset in (e) shows the quasi‐force imaging of the corresponding droplet where the experimental curve shown in (e) is along the blue line. The “distance 0” position is shown. f) Intensity, normalized quasi‐work, and normalized quasi‐force maps for a sample of interest along with a cross‐section for a representative droplet showing the contrast enhancement by the proposed framework. g) Reconstructed phase maps as a function of the regularization term *β*. All of the SEM images have the same scale bar of 10 µm.

Figure 2f shows the raw intensity, normalized quasi‐work, and normalized quasi‐force for another area of interest together with a cross‐section slice for a representative droplet. In the conventional intensity map, the signal from the droplet is comparable to the amplitude of the background noise. However, orders‐of‐magnitude improvement of the contrast via QN‐ESEM imaging can be seen in the normalized quasi‐work and normalized quasi‐force map (see the distribution of normalized quasi‐work along the cross‐section in Figure 2f). In addition to the clear physical meaning and strong connection with surface morphology, another feature of QN‐ESEM is the robustness against the random and systematic singularities, that is, a null value or a sharp jump in the raw intensity map that can be introduced either by the superposition of multiple random errors or by the instrument instability. To validate the robustness, we artificially created a null pixel at the center of the raw intensity map in Figure 2f (the area circled by a white box). The null pixel has no effects on *w*_∥_ and |**f**_∥_| imaging (Figure 2f), but it causes the phase reconstruction without regularization to completely fail (top left panel of Figure 2g). The huge difference between phase‐based enhanced ESEM and QN‐ESEM arises from the fact that the governing Equations (1) and (2) in QN‐ESEM includes local operators, while in the phase imaging, each pixel is not independent and thus localized defects will propagate throughout the entire image (see the Principle Section, Supporting Information). To address this instability, the phase reconstruction based approach typically adds a nonzero regularization constant *β* to the intensity image, ensuring the intensity at each pixel is strictly larger than zero.^[^ ^21^, ^25^ ^]^ However, this degrades imaging accuracy because of the lack of a general principle for the proper choice of *β*. If *β* is too small, image artifacts such as a pixel with a low intensity or a null value propagate and thereby mask the signals from the real droplets, while if *β* is too large, the droplet features are washed out (see the panels in Figure 2g for *β* = 4 × 10^−4^ and *β* = 1, respectively). Only when *β* is properly chosen will the phase map become reasonable (see the panel in Figure 2g for *β* = 2 × 10^−2^). To further demonstrate the robustness of QN‐ESEM, we also compared the normalized quasi‐work and normalized quasi‐force maps with the phase map for the case of a sharp jump in the raw intensity map. This jump can occur when the instrument experiences instability (see Figure S2, Supporting Information). Superior performance was obtained using QN‐ESEM in this case as well.

**Figure** **3** shows the dropwise condensation when the ESEM chamber pressure was elevated to 2500 Pa, which is more than two times higher than typical ESEM‐based condensation experiments.^[^ ^31^, ^32^, ^33^ ^]^ The probability of large‐angle electron‐molecule collision increases dramatically as the pressure in the chamber increases, which further lowers the spatial resolution, elevates background noise, and degrades the image contrast. The conventional ESEM intensity maps captured at five sequential time points fail to show the dynamics of condensing droplets (see representative frames in Figure 3a). As a comparison, we can find multiple condensing droplets in the normalized quasi‐work maps (Figure 3b). The dynamic growth of droplets (droplets 1–3 in Figure 3b) can be clearly seen in the normalized quasi‐work maps. As the droplets grew, the decrease of the gap between droplets 2 and 3 was observed (yellow arrow in Figure 3b); the droplets coalesced after 60 s. As discussed above, the maximum value for |**f**_∥_| occurs at the intersection of the detection plane and the droplet interface. For this reason, the |**f**_∥_|map provides a better sensitivity than the normalized quasi‐work map for observing the evolution of the liquid–vapor interface (Figure 3c). The dynamic growth of droplets recorded in a video in the same area of interest can be found in Movie S1, Supporting Information. A comparison of conventional ESEM and QN‐ESEM image contrast at different pressure conditions is presented in Figure S3, Supporting Information.

**Figure 3:**
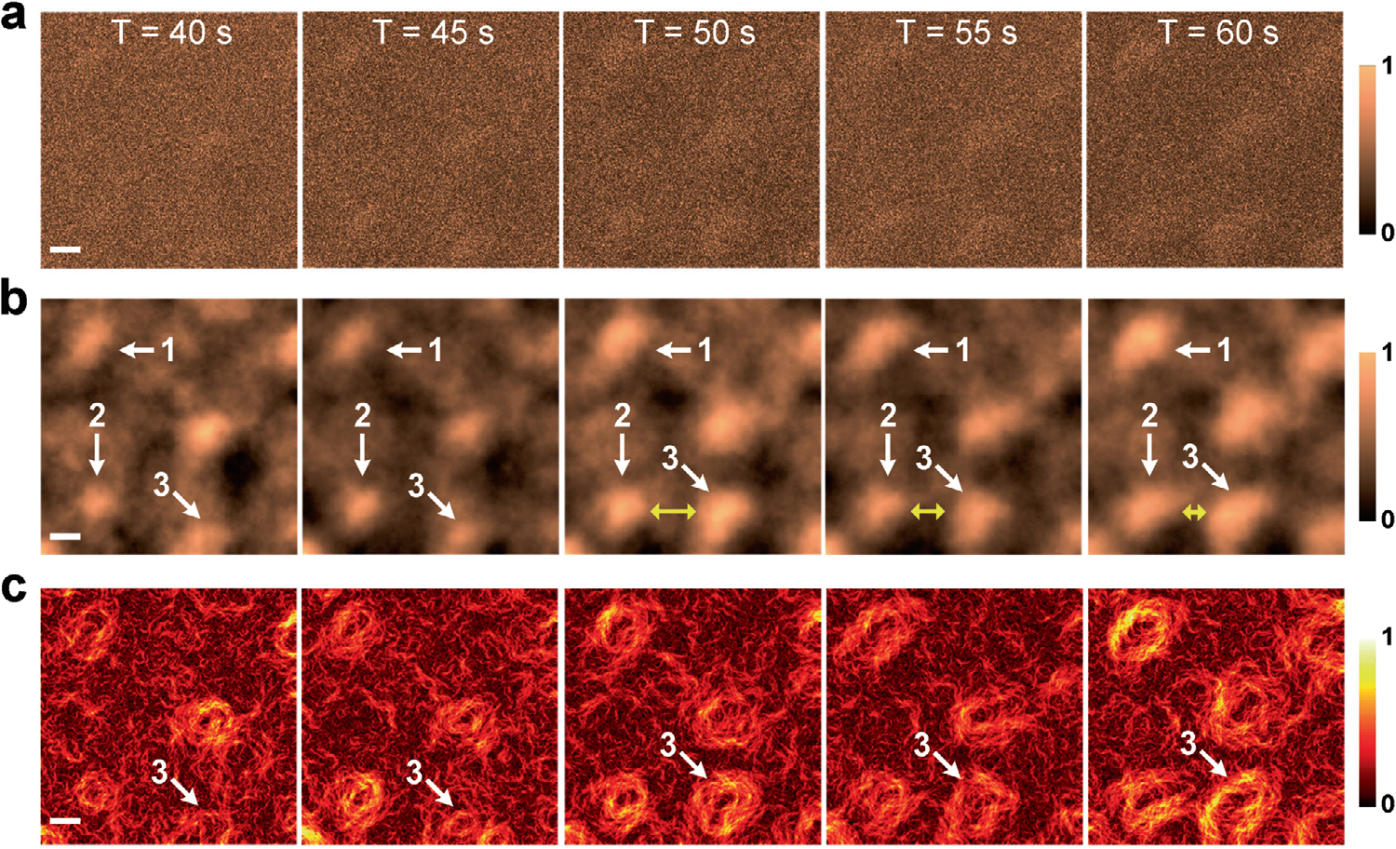
Normalized quasi‐work and quasi‐force recovered the dynamics of condensed droplets. a) Intensity, b) normalized quasi‐work, and c) normalized quasi‐force maps captured at five time points. The dynamic growth of several droplets as a function of time is marked with “1–3.” Starting from *T* = 50 s, we can observe the coalescence of droplets 2 and 3 from both the normalized quasi‐work and quasi‐force maps. All of the SEM images have the same scale bar of 10 µm.

We have demonstrated that QN‐ESEM is capable of reducing the noise from large‐angle electron‐molecule collision and enhancing the contrast which enables high quality images at more than double the operation pressure. Next, we discuss another degradation mechanism of conventional ESEM, specifically the one due to the random scattering from the nanotextured substrate and show the viability of QN‐ESEM for solving this challenge. Condensation on a copper oxide (CuO) nanostructured substrate was performed at 1300 Pa. A representative intensity image captured through conventional ESEM is presented in **Figure** **4**a, in which both the microscale and nanoscale features on the substrate degrades the image quality. Specifically, scratch‐like features were observed on the background (with the axis parallel to the dashed arrow shown in the bottom of Figure 4a); these are due to microscale trenches on the CuO substrate (the dark trench along with the marked axis in the SEM image of Figure 4b). In addition, the highly random CuO nanoblade structures lead to more scattering and further degrades the image quality. To demonstrate the degradation due to the trench and nanoblade structures, we performed Monte Carlo (MC) simulation of the electron microscope images,^[^ ^34^, ^35^, ^36^, ^37^, ^38^ ^]^ where the open‐source model (Casino v3.3) developed by Drouin et al. was used.^[^ ^36^ ^]^ To understand the electron–substrate interaction, we separate out the effect of the electron–gas interaction, that is, we simulate at 0 Pa. Modeling this complex and large‐scale electron–matter interaction for the substrate shown in Figure 4b is challenging because the de Broglie wavelength of electrons at 15 keV (0.083 nm) is many orders‐of‐magnitude smaller than the characteristic length scale (≈10‐100 µm) of the region of interests in Figure 4a. To simplify the problem, we separate our investigation by modeling the nanoblade structure‐induced and trench‐induced electron interactions, respectively. We first simulate a 20‐nm and a 40‐nm diameter droplet on a flat substrate (Figure 4c). Next, we build 25 square pyramids with 10‐nm bottom width and 18‐nm height to mimic the CuO nanoblades on the substrate (see the nanoblades beneath the droplet in the Figure 4d schematic). Because the dimensions of the pyramids are much larger than the electron wavelength, the simulation is adequate to qualitatively describe the scattering due to nanotexture. Figure 4d shows the simulated electron image of no droplet, a 20‐nm droplet, and a 40‐nm droplet on the nanoblade structures, which shows weak signal from the droplets since the noise from the nanoblade structures plays the dominant role. The simulated images of the droplets on the flat substrate (Figure 4c) shows better contrast than those on the nanoblade structures (Figure 4d). Better image contrast was also achieved for larger droplet size due to the increase of SCS. However, note the image quality for an experiment will likely be even worse because the nanoblades are randomly orientated (Figure 4b) rather than well‐arrayed (Figure 4d) as simplified in our simulation. Figure 4e shows the simulated electron image for the same droplets on a 20‐nm wide trench. When the droplet diameter is comparable to the trench width (see the electron images corresponding to *D* = 20 nm in Figure 4e), the contrast of the droplet is very poor because the scattering due to the droplet interface is comparable to that induced by the trench. When the droplet grows and its diameter becomes significantly larger than the trench width, it can be visualized via an increased contrast (see the image corresponding to *D* = 40 nm in Figure 4e). The insights gained from the MC simulation indicate that the wide trenches and nanoblade structures on the substrate are the other reasons for severely degraded imaging quality of relatively small size droplets. This explains the reason it is challenging for the conventional ESEM to characterize the early stages of nucleation, as shown in the dashed white boxes with markers 1–5 in Figure 4a. As a comparison, the normalized quasi‐work map *w*_∥_ from QN‐ESEM successfully recovered all of the hidden small size droplet (≤1 µm diameter) in boxes 1–5 of Figure 4f, in which three neighboring submicron size droplets located on microscale trenches were well‐resolved (see box 3 in Figure 4a,f). The background patterns due to trenches in the original ESEM image were removed, which is attributed to the operator property in Equation (2) that is robust against scattering‐invariant features on the substrate (see the Principle Section, Supporting Information). These droplets can also be seen in the normalized quasi‐force map |**f**_∥_| (Figure 4g), where strong scattering information were seen on the droplet interfaces. We should mention here that the proposed framework is repeatable and that the dwell time may be chosen within a reasonable range; see Figure S4, Supporting Information, and its description. Although the experiments implemented above were based on secondary electron images, our method also works for backscattering electron images, where the elastic scattering is more significant. See Figure S5, Supporting Information, and its description.

**Figure 4:**
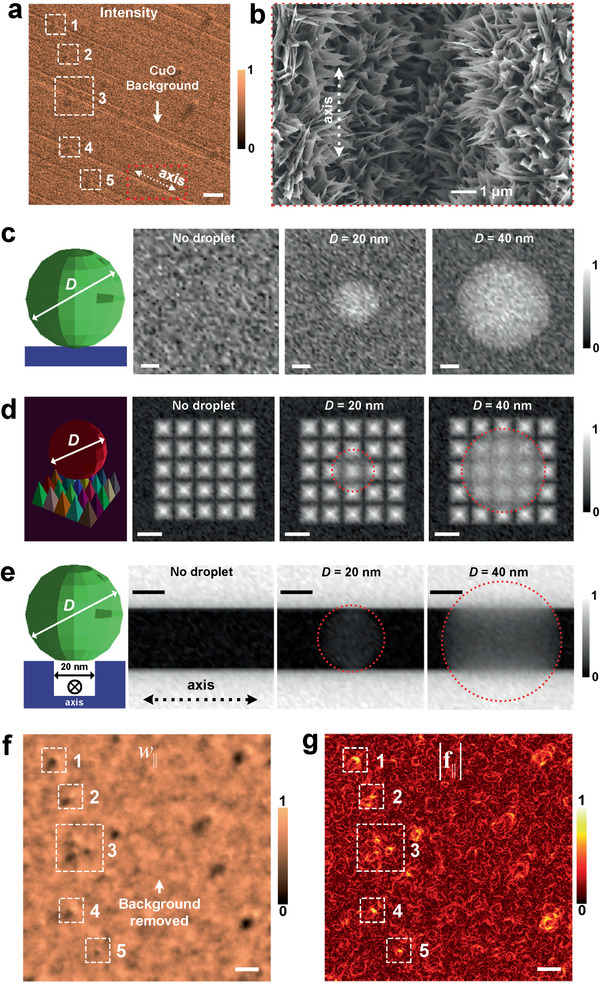
Conventional ESEM imaging for condensed droplets on a textured substrate and the disclosure for the underlying reasons of low contrast using Monte Carlo simulations. a) A representative ESEM intensity map. b) SEM image of an area of interest on the substrate (the red dotted box of [a]). Simulated SEM images for a water droplet on c) a flat CuO substrate, d) a pyramid array, and e) a 20‐nm wide trench, respectively. All of the simulated images in (c–e) have the same scale bar of 10 nm. Experimentally reconstructed f) normalized quasi‐work and g) normalized quasi‐force maps for the same area of interest in (a). The five dotted boxes highlight the successfully recovered droplets that are hardly visible in the conventional ESEM images in (a). (f) and (g) have the same scale bar of 10 µm.

In summary, we developed a new electron imaging framework called QN‐ESEM that outperforms conventional ESEM in characterizing materials dynamics in high‐pressure gaseous environments. The method does not need any modification to the hardware. Two new quantities, that is, the quasi‐work and quasi‐force were introduced, which describe the fundamentals of electron scattering at the sample interface. Various degradations of the electron image quality, due to both the large‐angle scattering induced by electron‐gas molecules collisions and the signal perturbations induced by random features on the substrate in conventional ESEM, can be effectively eliminated by the normalized quasi‐work and quasi‐force maps. We showed the normalized quasi‐force distribution is a function of the sample morphology, which enables a more quantitative analysis than previous electron imaging based on intensity and phase maps. In addition, a high robustness of QN‐ESEM against the singularities or sharp jumps due to instrument instabilities was demonstrated. By leveraging the ultrahigh sensitivity and robustness of QN‐ESEM, imaging the condensation at high‐pressure gaseous chamber (up to 2500 Pa) and detecting submicron diameter droplets on the nanotextured substrate became possible. Although the experimental demonstration was performed on condensing droplets, we believe the general framework of QN‐ESEM paves a path to investigate various dynamic behavior, including the evolution of hydrated or biological samples, nucleation, aggregation, and chemical reactions in an extreme environment with unprecedented simplicity and effectiveness.

## Experimental Section

##### Fabrication of CuO Nanotextured Surface

The chemical and plasma cleaned copper substrate was immersed into a hot alkaline solution, which was maintained at ≈95 °C. The alkaline solution consisted of NaClO_2_, NaOH, Na_3_PO_4_·12H_2_O, and DI water (3.75:5:10:100 wt%). CuO nanoblades formed due to the surface reaction.^[^ ^39^, ^40^ ^]^ Next, a less than 100‐nm thick hydrophobic layer was conformally coated on the surface by the company P2i.

##### ESEM Experiment

The substrate was mounted on a temperature controlled stage. The temperature of saturated vapor in the ESEM chamber (Zeiss EVO 50 scanning electron microscope) was initially set to the same as the temperature controlling stage. Next, the vapor temperature was elevated by Δ*T* to initiate condensation, where Δ*T* ≈ 1 °C was applied for this study and Δ*T* was known as the subcool temperature between the substrate and the saturated vapor. Videos were taken after the condensation occurred.

##### Statistical Analysis

All data statistical analyses were performed using Origin software (version 2018b, Origin Lab Inc., USA). Statistical analysis was compiled on the means of the data obtained from at least three independent experiments with three replicates in each case. All data were expressed as the means ± standard error.

## Conflict of Interest

The authors declare no conflict of interest.

## Supporting information

Supporting Information

Supplemental Movie 1

Supplemental Movie 2
