# Supplementary material for: Quasi‐Newtonian Environmental Scanning Electron Microscopy (QN‐ESEM) for Monitoring Material Dynamics in High‐Pressure Gaseous Environments
Source: Adv Sci (Weinh). 2020 Aug 18;7(19):2001268. doi: 10.1002/advs.202001268 (PMC7539182; doi:10.1002/advs.202001268)
Supplement: Supplementary file 1 — Supporting Information [file ADVS-7-2001268-s001.pdf]

© 2020 Wiley-VCH GmbH

ADVANCED SCIENCE
Open Access

Supporting Information

for *Adv. Sci.*, DOI: 10.1002/advs.202001268

Quasi-Newtonian Environmental Scanning Electron Microscopy (QN-ESEM) for Monitoring Material Dynamics in High-Pressure Gaseous Environments

Jinlong Zhu, Lenan Zhang, Xiangyu Li, Kyle L. Wilke, Evelyn N. Wang, and Lynford L. Goddard**

Supporting Information

Quasi-Newtonian Environmental Scanning Electron Microscopy (QN-ESEM) for Monitoring Material Dynamics in High-Pressure Gaseous Environments

Jinlong Zhu, Lenan Zhang*, Xiangyu Li, Kyle L. Wilke, Evelyn N Wang†, and Lynford L. Goddard†*

*Equal contribution to this work
†Correspondence at: lgoddard@illinois.edu and enwang@mit.edu

Dr. Jinlong Zhu*
Department of Electrical and Computer Engineering, University of Illinois at Urbana-Champaign, Urbana, IL 61801, USA

Mr. Lenan Zhang*
Department of Mechanical Engineering, Massachusetts Institute of Technology, Cambridge, MA 02139, USA

Dr. Xiangyu Li
Department of Mechanical Engineering, Massachusetts Institute of Technology, Cambridge, MA 02139, USA

Dr. Kyle L. Wilke
Department of Mechanical Engineering, Massachusetts Institute of Technology, Cambridge, MA 02139, USA

Prof. Evelyn N. Wang†
Department of Mechanical Engineering, Massachusetts Institute of Technology, Cambridge, MA 02139, USA

Prof. Lynford L. Goddard†
Department of Electrical and Computer Engineering, University of Illinois at Urbana-Champaign, Urbana, IL 61801, USA

This file includes:

Principle Section and Extended Data

Supporting Information Figure S1 and S2

Captions for Supporting Information Movies 1 and 2

Supporting Information References

Principle Section:

The transverse mechanical force in a 2D imaging system

The field equation of a paraxial beam propagating along the *z*-direction satisfies

$$E(\vec{\mathbf{r}})\exp\left[ik_z(\vec{\mathbf{r}})z\right] = u(\vec{\mathbf{r}})\exp\left[ik_z(\vec{\mathbf{r}})z + i\varphi(\vec{\mathbf{r}})\right], \tag{S1}$$

where $u(\vec{\mathbf{r}})$ and $\varphi(\vec{\mathbf{r}})$ are the spatial-variant transverse amplitude and phase of the field $E(\vec{\mathbf{r}})$. $k_z(\vec{\mathbf{r}})z$ denotes the phase induced by the propagation of wave along *z*-direction. It is well-known that photons carry momentum; the momentum flux $\mathbf{g}(\vec{\mathbf{r}})$ in a beam of electromagnetic wave is proportional to the intensity (defined as $\left|E(\vec{\mathbf{r}})\right|^2$). Because in a paraxial beam $k_z(\vec{\mathbf{r}})$ is approximately a constant ($\left|k_z(\vec{\mathbf{r}})\right| \approx |\mathbf{k}| = k$), it is not difficult to conclude that the transverse momentum flux, $\mathbf{g}_{\parallel}(\vec{\mathbf{r}})$, is a function of the transverse phase, $\varphi(\vec{\mathbf{r}})$, i.e.,

$$\mathbf{g}_{\parallel}(\vec{\mathbf{r}}) = \frac{k}{n_{\mathrm{b}}\mu_0 v}\left|E(\vec{\mathbf{r}})\right|^2 \nabla_{\parallel}\varphi(\vec{\mathbf{r}}), \tag{S2}$$

where n_{b}, μ_0, and v denote the background index, the permeability of vacuum, and the speed of wave in vacuum. Here n_{b} and μ_0 are constants that are meaningful in optics; however, we show in the following that these constants can be eliminated to provide an analogy to the electron wave function. For a non-magnetic small object positioned in the paraxial beam, the momentum of the wave will result in the movement of the object, indicating the existence of mechanical force and work (provided the object is freely moveable). In optics, the mechanical force primarily consists of the scattering force because the trapping force is negligible due to the adoption of paraxial illumination. Therefore, the transverse component of the mechanical

force exerteded on the object arises from the transverse momentum flux of wave. Mathematically, the transverse force has a concise form, i.e.,

$$\mathbf{F}_{\parallel}(\vec{\mathbf{r}}) = \frac{k\sigma}{\mu_0}\left|E(\vec{\mathbf{r}})\right|^2 \nabla_{\parallel}\varphi(\vec{\mathbf{r}}), \tag{S3}$$

where σ is the extinction cross-section of the small object. Note that for a given illumination, σ is a constant; thus, $\mathbf{F}_{\parallel}$ only depends spatially on E and φ .

Measuring the mechanical force and work in a 2D imaging system

The field equation of a paraxial beam propagating along the z-direction satisfies the paraxial Maxwell equation: $\left[2ik\nabla_{\perp} + \nabla_{\parallel}\right]E(\vec{\mathbf{r}}) = 0$. By inserting Equation S1 into the paraxial equation, we can derive an amplitude-phase coupling equation

$$k\nabla_{\perp}\left|E(\vec{\mathbf{r}})\right|^2 = -\nabla_{\parallel}\left|E(\vec{\mathbf{r}})\right|^2 \cdot \nabla_{\parallel}\varphi(\vec{\mathbf{r}}) - \left|E(\vec{\mathbf{r}})\right|^2 \Delta_{\parallel}\varphi(\vec{\mathbf{r}}), \tag{S4}$$

where $\Delta_{\parallel}$ is the transverse Laplacian and $\nabla_{\perp}$ denotes the longitudinal derivative. Equation S4 can be simplified via some algebraic operations such that it has a concise form

$$k\nabla_{\perp}\left|E(\vec{\mathbf{r}})\right|^2 = -\nabla_{\parallel}\cdot\left[\left|E(\vec{\mathbf{r}})\right|^2 \nabla_{\parallel}\varphi(\vec{\mathbf{r}})\right]. \tag{S5}$$

By comparing the term inside the square bracket on the right-hand side (RHS) of Equation S5 with the RHS of Equation S3, we can find that the left-hand side (LHS) of Equation S5 is proportional to the transverse divergence of the transverse scattering force $\mathbf{F}_{\parallel}$ because $\frac{k\sigma}{\mu_0}$ is a constant term. Therefore, Equation S5 can be cast into a physically meaningful form

$$\frac{k^2\sigma}{\mu_0}\nabla_{\perp}\left|E(\vec{\mathbf{r}})\right|^2 = -\nabla_{\parallel}\cdot\mathbf{F}_{\parallel}(\vec{\mathbf{r}}), \tag{S6}$$

where $\nabla_{\perp}\left|E(\vec{\mathbf{r}})\right|^2$, which is the longitudinal derivative of irradiance and is a measurable quantity in a microscopy system. From Newton's law of motion, we know that the mechanical work and force is governed by $\nabla_{\parallel}W_{\parallel}(\vec{\mathbf{r}}) = \mathbf{F}_{\parallel}(\vec{\mathbf{r}})$, where $W_{\parallel}(\vec{\mathbf{r}})$ is the transverse mechanical work. Equation S6 can be reformulated as

$$k\nabla_{\perp}\left|E(\vec{\mathbf{r}})\right|^2 = -\Delta_{\parallel}w_{\parallel}(\vec{\mathbf{r}}) \qquad \text{(S7)}$$

by introducing the normalized term $w_{\parallel}(\vec{\mathbf{r}}) = \frac{W_{\parallel}(\vec{\mathbf{r}})\mu_0}{k\sigma}$, which is termed as normalized quasi-work in this paper. Similarly, the normalized quasi-force is defined as $\nabla_{\parallel}w_{\parallel}(\vec{\mathbf{r}}) = \mathbf{f}_{\parallel}(\vec{\mathbf{r}})$. The normalization is valid because k, σ, and μ_0, again, are constants that do not impact the spatial properties of $W_{\parallel}(\vec{\mathbf{r}})$ and $\mathbf{F}_{\parallel}(\vec{\mathbf{r}})$.

Features of quasi-force and quasi-work reconstruction in scanning electron microscopy

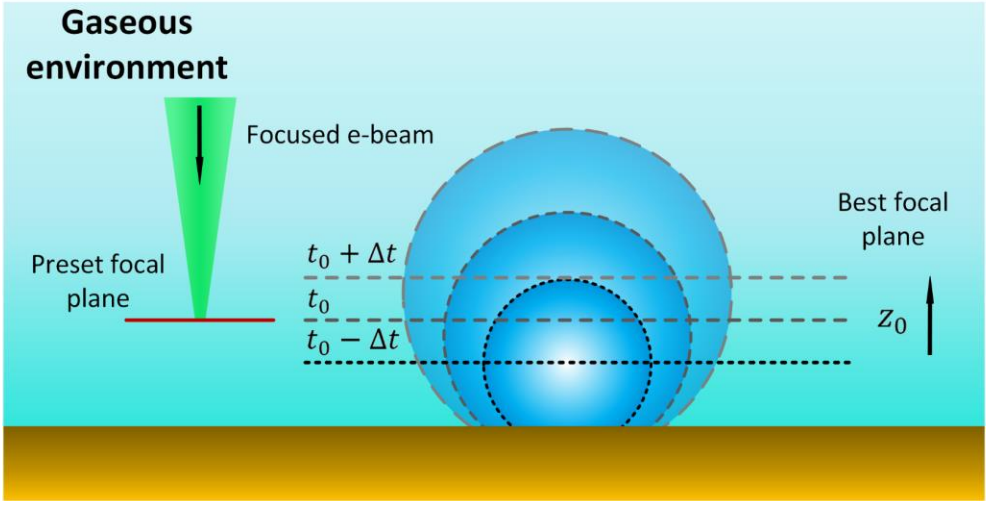

Figure S1. Schematic showing the dynamic changing of best focal plane z_0 as a condensed droplet grows from second t_0-Δt to t_0+Δt. The distance between the tip of the focused e-beam and the substrate, known as the position of the preset focal plane, is a constant during the horizontal scanning, leading to an automatic depth scanning along the optical axis to collect both the in-focus and out-of-focus information.[1]

Applying Equation S7 to an SEM is non-trivial because the electron-matter-interaction induced by a high-energy electron beam is nonlinear and polychromatic (i.e., the backscattered and secondary electrons correspond to longer de Broglie wavelengths compared to the excitation source due to energy loss). However, we can still apply Equation S7 to reconstruct $w_{\parallel}(\vec{\mathbf{r}})$ and $\mathbf{f}_{\parallel}(\vec{\mathbf{r}})$ with the knowledge that no ideally monochromatic source exists in practice. Therefore, the reconstructed $w_{\parallel}(\vec{\mathbf{r}})$ and $\mathbf{f}_{\parallel}(\vec{\mathbf{r}})$ are "polychromatic quantities."

For a dynamic object, the corresponding irradiance $|E|^2$ is time-variant. For example, for the condensed droplet, the center (best focal plane) of a droplet rises due to the dynamic growth during condensation, leading to a "sample-induced" variation in the irradiance along the longitudinal direction. Therefore, $\nabla_{\perp}|E|^2$ can be calculated by a simple second-order difference $\nabla_{\perp}|E(\vec{\mathbf{r}})|^2 = \left[|E(\vec{\mathbf{r}})_{z_0+\Delta z}|^2 - |E(\vec{\mathbf{r}})_{z_0-\Delta z}|^2 \right] / 2\Delta z$, where $|E(\vec{\mathbf{r}})_{z_0+\Delta z}|^2$ and $|E(\vec{\mathbf{r}})_{z_0-\Delta z}|^2$ are the measured irradiance at $z_0+\Delta z$ and $z_0+\Delta z$ planes, respectively.

Matrix-based solver for $w_{\parallel}(\vec{\mathbf{r}})$ ***and*** $\mathbf{f}_{\parallel}(\vec{\mathbf{r}})$

By replacing the left-hand side of Equation S7 with a constant matrix **G**, Equation S7 is a Poisson equation, which can be solved in matrix form **TW** + **WT** = **G**, by introducing a tri-diagonal matrix **T** with non-zero elements [1, −2, 1] in the middle rows and [-1, 1] and [1, −1] in the first and last rows, respectively. Here **W** is normalized quasi-work matrix with each entry $\mathbf{W}_{ij} = w_{\parallel}(x_i, y_j)$. The dimensions of **W** correspond to the number of scanning points in one scanning. Because **T** is a real Hermitian matrix, we can diagonalize it by using a unitary matrix **Q** such that **TW** + **WT** = **G** can be reformulated into a solvable form

$$\mathbf{SK} + \mathbf{KS} = \mathbf{H}, \tag{S8}$$

where $\mathbf{S} = \mathbf{Q}^*\mathbf{T}\mathbf{Q}$ is diagonal, $\mathbf{K} = \mathbf{Q}^*\mathbf{W}\mathbf{Q}$, and $\mathbf{H} = \mathbf{Q}^*\mathbf{G}\mathbf{Q}$. Note that **T**, **Q**, and **G** are known matrices. The elements of **K** can be computed directly by

$$K(i, j) = \frac{H(i, j)}{S(i,i) + S(j, j)}, \tag{S9}$$

after which the unknown normalized quasi-work matrix **W** is obtained via $\mathbf{QKQ}^*$. The normalized quasi-force matrix **F** with the entries $\mathbf{F}_{ij} = \mathbf{f}_{\parallel}(x_i, y_j)$ can be readily obtained via the transverse gradient operation. To address the possible issue of instability arising from small eigenvalues $S(i, i)$ and measurement errors, the regularization method (for instance, Tikhonov regularization) can be introduced.[2,3]

Operator properties in QN-SEM and the comparison with phase reconstruction

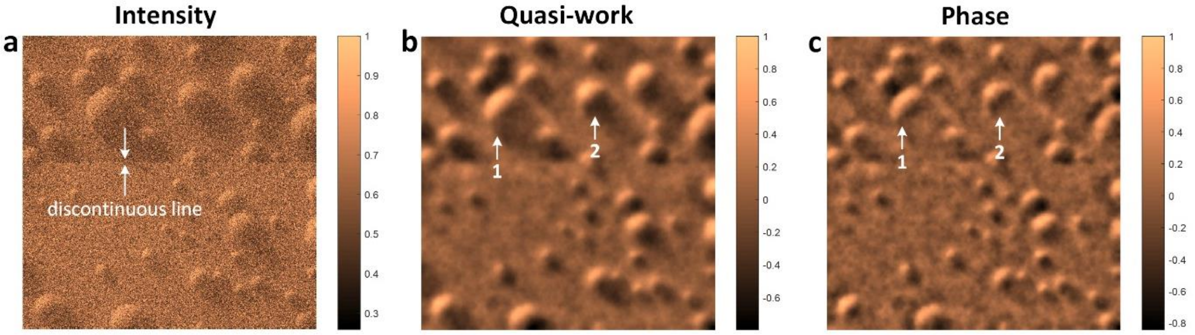

Figure S2. The a) measured intensity map, b) normalized quasi-work map, and c) phase map. There is a discontinuous line in the intensity map (marked by a pair of arrows in a)) due to an instrument error. The condensing experiment was implemented at 1500 Pa on a silicon substrate.

The gradient and Laplacian operators in Equation S7 are local ones. By local, we mean that applying the operator does not enable a local feature to have significant impact on the other areas of the image. This indicates that if there are "abnormal pixels" in an SEM image such as those caused by system instability or measurement error (for example, a zero-intensity pixel), the local abnormality will not propagate to the other areas in the image when reconstructing normalized quasi-work and quasi-force. However, in the matrix-based solver (Equations S8

and S9), the introduction of the global operator matrices such as **Q** and $\mathbf{Q}^*$ may alter the local properties and result in a spread of a local abnormality. The application of regularization techniques suppresses this phenomenon and maintains the high-contrast in the normalized quasi-work and quasi-force maps. However, in the phase-based SEM imaging framework,[1] the abnormality of the problem is further deteriorated because of dividing $\nabla_{\parallel} w_{\parallel}(\vec{\mathbf{r}})$ by the raw irradiance $\left|E(\vec{\mathbf{r}})\right|^2$ (see more details in ref. [4]). To alleviate this abnormality, we can, for instance, add a nonzero value to the "abnormal pixels" or to all the pixels. But because the levels of systematic errors are difficult to know, determining a reasonable correction value requires a trial-and-error effort in practice (see Figure 2g). This issue in phase reconstruction in turn reflects the fact that quasi-work and quasi-force reconstruction is more robust to perturbations in the SEM imaging process than phase reconstruction.

To further demonstrate the advantage of the proposed QN-SEM over phase-based ESEM, we consider another case of "abnormal pixels" where a discontinuous line exists on several frames of a video when recording the growth dynamics of condensed droplets in a conventional ESEM. This phenomenon is commonly seen in ESEM due to the mismatch between the recording rate of the detector and the scanning frequency of the electron beams. As shown in Figure S2a, there is a blurry horizontal line (indicated by two arrows) in the intensity map captured at 1500 Pa. The reconstructed normalized quasi-work map, as shown in Figure S2b, not only successfully recovered the inclined illumination-induced shadow of each droplet, but also maintained the binocular vision with a high fidelity. However, using phase reconstruction, the images of droplets were severely distorted—there were no more shadows and the droplets are no longer spherical. See two representative droplets marked by "1" and "2" in the phase map and compare them with those in the quasi-work map. This result demonstrates again that the proposed framework outperforms the phase reconstruction method in resisting system errors.

Quasi-work and quasi-force images as a function of pressure

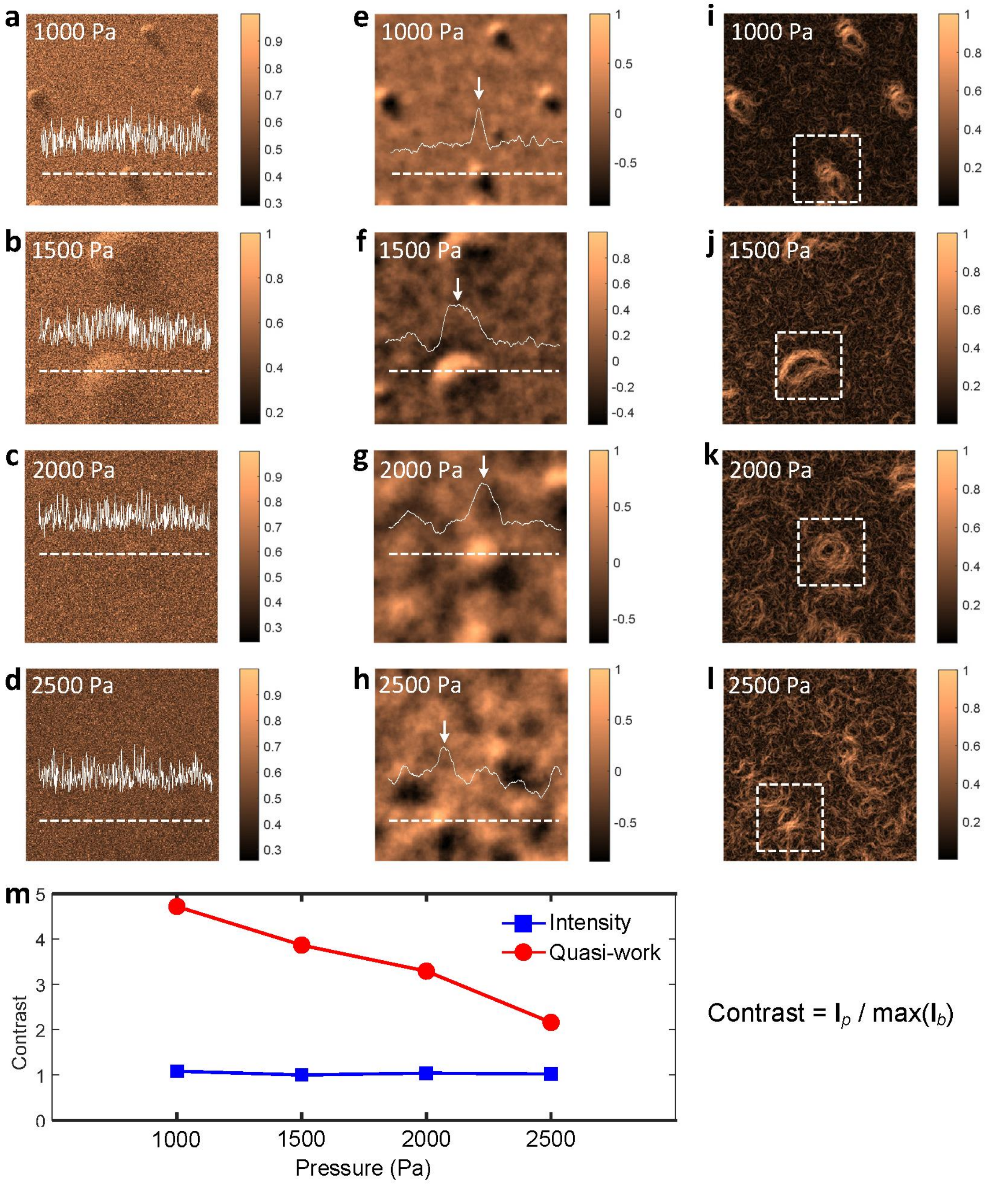

Figure S3. Reconstructed quasi-work and quasi-force images as a function of pressure. a-d) raw intensity images, e-h) quasi-work images, and i-l) quasi-force images. m) Contrast curves of the selected droplet for quasi-work (red circles) images and intensity (blue squares) images. The contrast is defined as the ratio of the strength of the peak signal ($\mathbf{I}_p$) from a droplet to the peak signal [max($\mathbf{I}_b$)] of the background noise.

To further demonstrate the advantage of the proposed framework, we reconstructed quasi-work and quasi-force images from raw intensity images captured at 1000 Pa, 1500 Pa, 2000 Pa, and 2500 Pa, respectively. As depicted in Figure S3e-h and i-l, quasi-work and quasi-force imaging precisely recovered the size and morphology of each droplet, where the image contrast was significantly improved, and the background noise was successfully suppressed. We next study the intensity and reconstructed quasi-work distribution (the white solid curves) along a line (see the white dotted lines) crossing the center of a droplet in Figure S3a-h. Apparently, the intensity signal is comparable to the intensity of background noise, making it difficult to distinguish the droplets using the intensity distribution. In contrast, one can easily find a peak that corresponds to the droplet from the quasi-work distribution. To quantitatively analyze the enhancement, we define the image contrast as the ratio of the strength of the peak signal ($\mathbf{I}_p$) from a droplet to the peak value [max($\mathbf{I}_b$)] of background noise, i.e.,

$$Contrast = \frac{\mathbf{I}_p}{\max(\mathbf{I}_b)}. \qquad \text{(S10)}$$

According to Eq. (S10), a smaller *Contrast* indicates a lower image quality and implies that it is more difficult to find droplets. Figure S3m shows the contrast of the intensity and quasi-work as a function of pressure. The contrast of intensity always stayed near 1, indicating that the peak signal of the droplet was comparable to that of the background noise. Using the quasi-work reconstruction, the contrast was improved by a factor of five at 1000 Pa and decayed with pressure because of increased random electron scattering at higher pressure. However, an enhancement of approximately two in contrast at 2500 Pa was still achieved, which is enough for us to retrieve the droplets from the quasi-work images. Note the contrast analysis was only performed on the raw intensity and quasi-work images. The quasi-force exhibits peaks at the boundaries of the droplet, which makes it difficult to define the contrast

in a way that can be directly compared with the contrast of the intensity and quasi-work images.

Repeatability of the proposed framework for different dwell times

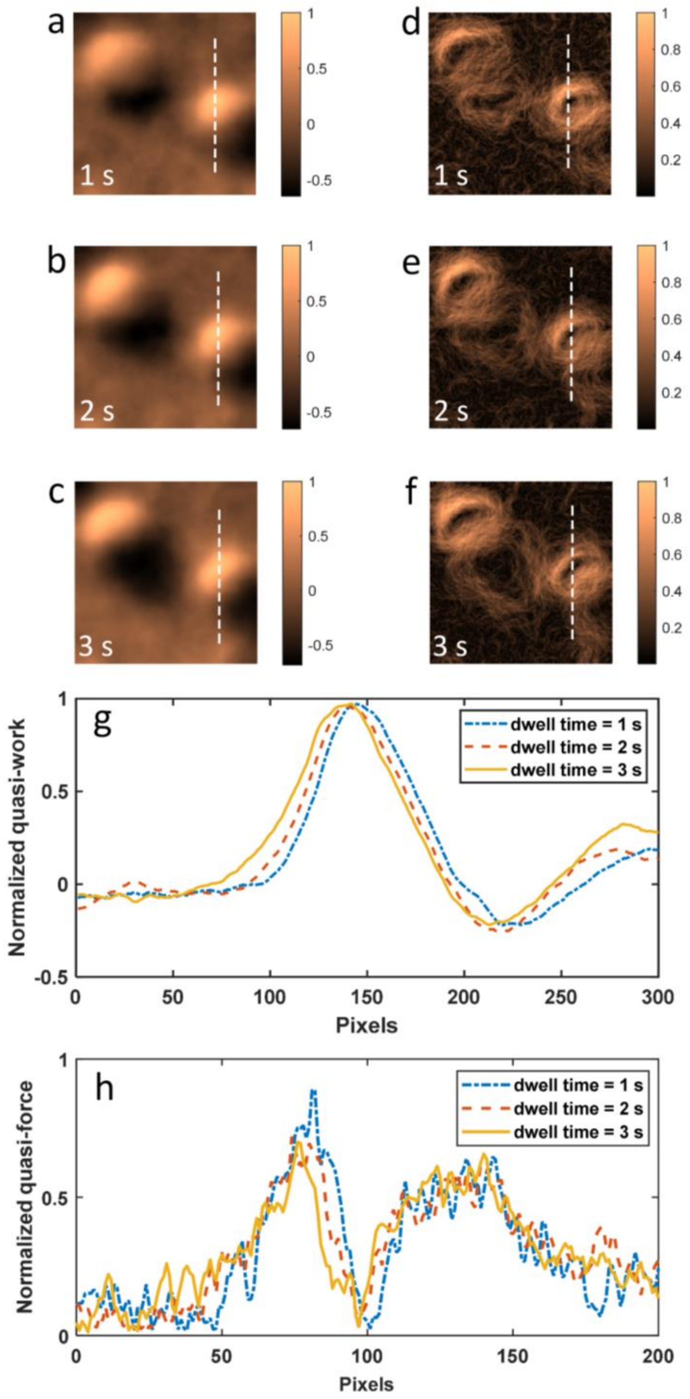

Figure S4. Reconstructed quasi-work and quasi-force images under different dwell times. a-c) Quasi-work images and d-f) quasi-force images. The first, second, and third rows correspond to the reconstructed quasi-quantities with the dwell time set as 1 s, 2 s, and 3 s, respectively.

The quasi-work and quasi-force distribution along a line crossing the center of a representative droplet is presented in g) and h), respectively.

To demonstrate the repeatability, we reconstruct the quasi-work and quasi-force for different dwell times. As presented in Figures S4a-c, the reconstructed quasi-work maps are visually indistinguishable. A quasi-work distribution along a line crossing the center of a representative droplet (see the dotted lines in Figure S4a-c) shows that the morphologies have only minor difference due to the different noise distribution and levels in the frames captured at different time. Through the quasi-force images, we can more clearly observe the difference induced by different dwell time, but the morphologies of droplets still agree well. A quasi-force distribution along the same line crossing the center of a representative droplet clearly validates that different dwell times do not change the morphologies of droplets, which means the quasi-force is repeatable as well. (see Figure S4h). The stronger fluctuations in the curves demonstrates that quasi-force is more sensitive to the environments than quasi-work. Here, we should mention that the dwell time should not be arbitrarily large; this is because the three adjacent frames (used to reconstruct the quasi-work and quasi-force) captured at a very large dwell time may contain entirely different droplets due to the significant growth and coalescence of droplets.

Effectiveness of the proposed framework on backscattering electron images

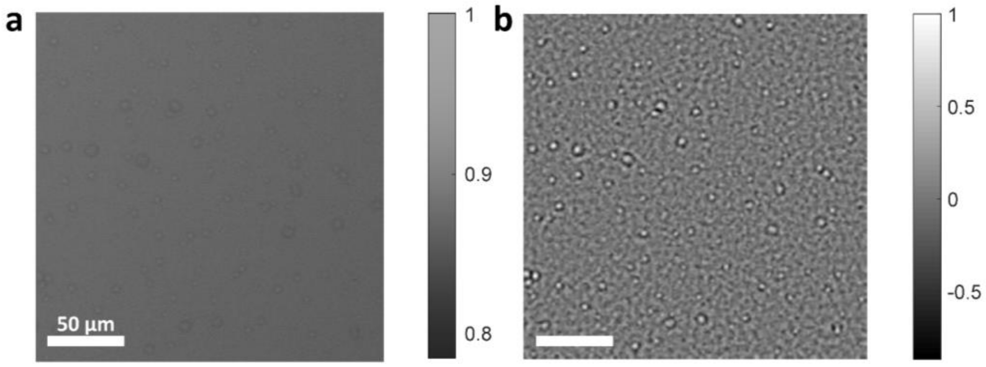

Figure S5. Reconstructed quasi-work image for backscattering electron image. a) Raw ESEM image of condensing droplet using the backscattered electron detector, where the elastically

backscattered electrons are dominant during the imaging process. The raw ESEM image has very low contrast due to the electron-gas scattering at high pressure (1000 Pa). b) Retrieved quasi work image using the developed approach where the image quality was significantly improved, and a few micron size droplets were well resolved.

Our imaging framework is relatively weakly dependent on the elastic and inelastic scattering conditions. To demonstrate this point, we further imaged the droplet condensation using the backscattering electron detector in ESEM (Figure S5), where the elastic scattering is more significant because the backscattered electron has larger energy than the secondary electron [3,4]. Similar to the results observed using the secondary electron detector, the raw ESEM image (Figure S5a) has very low contrast due to the intense electron-gas molecule scattering at high pressure (1000 Pa). However, the image quality can be significantly improved using the "quasi work" treatment as shown in Figure S5b, where a number of droplets that are only a few microns in size can be well resolved. Considering similar enhancement can be achieved using both the backscattered electron and the secondary scattered electron imaging, we believe the imaging technique developed in this work is useful and robust.

Captions for Supporting Information Movies 1 and 2

Figure caption of Movie 1. Comparison among intensity, quasi-work, and quasi-force maps. The condensing experiment was implemented at 1300 Pa on a silicon substrate.

Figure caption of Movie 2. Comparison among intensity, quasi-work, and quasi-force maps. The condensing experiment was implemented at 2500 Pa on a silicon substrate.

Supporting Information References

[1] L. Zhang, J. Zhu, K. L. Wilke, Z. Xu, L. Zhao, Z. Lu, L. L. Goddard, E. N. Wang, *ACS Nano* **2019**, *13*, 1953.

[2] J. Zhu, R. Zhou, L. Zhang, B. Ge, C. Luo, L. L. Goddard, *Opt. Express* **2019**, *27*, 6719.

[3] J. C. Petruccelli, L. Tian, G. Barbastathis, *Opt. Express* **2013**, *21*, 14430.

[4] J. Zhu, Y. Liu, X. Yu, R. Zhou, J.-M. Jin, L. L. Goddard, *Nano Lett.* **2019**, 19, 5347.
